# Carpal Tunnel Syndrome Associated with Immune Checkpoint Inhibitors

**DOI:** 10.3390/jpm13091340

**Published:** 2023-08-30

**Authors:** Alexander Yakobson, Keren Rouvinov, Aharon Y. Cohen, Iris Goldstein, Omar Abu Saleh, Adam Solomon, Yulia Dudnik, Walid Shalata

**Affiliations:** 1 The Legacy Heritage Cancer Center & Larry Norton Institute, Soroka Medical Center, Beer Sheva 84105, Israel; 2 Faculty of Health Sciences, Ben-Gurion University of the Negev, Beer Sheva 84105, Israel; 3 Department of Neurology, Soroka University Medical Center, Faculty of Health Sciences, Ben Gurion University, Beer Sheva 84105, Israel; 4 Department of Dermatology and Venereology, The Emek Medical Centre, Afula 18341, Israel; 5 Medical School for International Health and Sciences, Ben-Gurion University, Beer-Sheva 84105, Israel

**Keywords:** PD1 inhibitor, PDL-1 inhibitor, carpal tunnel syndrome (CTS), non-small-cell lung cancer (NSCLC), renal cell carcinoma (RCC), malignant melanoma (MM), immune-related adverse events (irAEs)

## Abstract

Immune checkpoint inhibitors (ICIs) have transformed the therapeutic approach to diverse malignancies, leading to substantial enhancements in patient prognosis. However, along with their benefits, ICIs also increase the incidence of immune-related adverse events (irAEs). In the present paper, we highlight four cases of carpal tunnel syndrome (CTS) as an uncommon manifestation of toxicity induced by ICIs. Although diagnosed with different malignancies, the patients were undergoing ICI therapy when they developed CTS-consistent side effects accompanied by severe neuropathy. Prompt treatment with corticosteroids, intravenous immunoglobulins, or methotrexate resulted in complete symptomatic relief for all patients. This article therefore emphasizes the importance of recognizing and managing rare adverse events associated with ICI use to ensure optimal patient care.

## 1. Introduction

Immune checkpoint inhibitors (ICIs) have revolutionized treatment approaches and prognoses for several types of malignancies. They have emerged as a significant breakthrough in cancer therapeutics, establishing new standards of care across various treatment settings [1]. In the neoadjuvant setting, ICIs have been explored as a preoperative treatment to reduce tumor size and increase the likelihood of successful surgical resection. This approach aims to optimize response to treatment, potentially improving the chances of long-term remission or cure [2,3].

In the adjuvant setting, ICIs have demonstrated efficacy in reducing the risk of disease recurrence following surgery or other first-line treatments. By enhancing the capacity of the immune system to identify and eliminate residual malignant cells, ICIs have shown the potential to improve long-term patient outcomes [2,4,5,6].

In the metastatic setting, ICIs have transformed the treatment landscape by providing durable responses and improved survival rates for patients with advanced-stage cancers. They have therefore become a cornerstone of therapy in various malignancies, such as melanoma, lung cancer, renal cell carcinoma, colorectal cancer, cervical cancer, breast cancer, and others [2,7,8,9,10,11,12].

The effectiveness of ICIs lies in their ability to target immune checkpoints and disrupt the mechanisms that cancer cells utilize to evade immune recognition and targeted programmed cell death. By blocking transmembrane receptors of the immune checkpoint apparatus, including cytotoxic T-lymphocyte-associated antigen-4 (CTLA-4), programmed cell death protein (PD-1), and its ligand (PD-L1), ICIs unleash the adaptive cellular immune system, enabling it to mount a robust and sustained anti-tumor response [13,14]. However, adverse events characteristic of autoimmunity were initially documented in animal models secondary to the immune checkpoint blockade [15,16]. 

The relationship between gender and the risk of immune-related adverse events (irAEs) may hinge on the specific class of ICIs utilized. In instances involving inhibition of PD-1/PD-L1 (across various cancers, including melanoma and lung cancer), men exhibited an elevated susceptibility to pulmonary toxicity. Conversely, women faced an elevated risk of irAEs associated with CTLA-4 inhibitors (in the context of melanoma). Notably, the nature of irAEs also appears to diverge between genders. In particular, women tend to experience a higher incidence of irAEs related to endocrinopathies, particularly thyroid dysfunction, while demonstrating fewer occurrences of neurological, dermatological, and vascular irAEs compared to men. Retrospective assessments of cardiac events in ICI therapy have highlighted distinct gender-based patterns. Specifically, men were found to be at an increased risk of irAEs such as myocarditis, pericarditis, arrhythmias, as well as coronary artery disease and myocardial infarction [13,16,17,18,19,20].

Studies comparing irAEs in malignant melanoma (MM) patients noted an increased incidence of all-grade irAEs in the gastrointestinal (GI) system, such as colitis and diarrhea, and skin, such as rash and pruritus, compared to patients with non-small-cell lung cancer (NSCLC). Additionally, compared to patients with renal cell carcinoma (RCC), these patients experience a greater frequency of irAEs in the GI system, such as diarrhea; skin; endocrine system, such as hyperthyroidism or hypothyroidism; and musculoskeletal system, such as arthralgia. Moreover, they manifest a lower incidence of irAEs in the lungs, such as pneumonitis and dyspnea, compared to both NSCLC and RCC patients. The onset of irAEs tends to occur later in MM patients compared to lung cancer patients, with an average duration of almost 5 months and 2 months, respectively [16].

In myeloid malignancies, such as relapsed/refractory acute myeloid leukemia (AML) and myelodysplastic syndromes (MDS) treated with ICI, high-grade events (at least Grade 3) commonly involve the skin (15%) and liver (11%) [21]. It was reported that fatal adverse events (FAEs) associated with pembrolizumab were highest among breast cancer patients (3.2–1%), followed by NSCLC (2%), urothelial carcinoma (0.8%), and MM (0.2%) [22]. Combining chemotherapy with ICIs increased the risk of FAEs from 0.7% to 7%; however, there was no significant difference between the ICI and chemotherapy groups. Infectious complications were the most common cause of FAEs, followed by cardiac toxicity and pneumonitis. Another study found that non-lung-cancer patients, such as those with malignant melanoma and RCC, had a lower risk of hospitalization due to irAEs [23]. Neurological irAEs have an incidence ranging from 1% to 12% and are associated with a higher fatality rate compared to other adverse events. The lack of standardized disease definitions and precise phenotyping contributes to the misclassification of syndromes, which hinders the development of evidence-based treatments and translational research in this field [24]. 

Carpal tunnel syndrome (CTS) is the most prevalent nerve compression or entrapment syndrome affecting the upper extremity. Approximately 5% of individuals per year in the United States are estimated to be affected, with a higher incidence in women compared to men. In the majority of CTS cases, the underlying cause remains unknown [25]. However, certain systemic conditions, including diabetes mellitus, rheumatic diseases, such as rheumatoid arthritis, and patients with renal failure may have an Increased susceptibility to developing CTS. As the diagnosis of CTS is primarily clinical, it is suspected when patients manifest classical symptoms including nocturnal pain, numbness, tingling sensations, and neuropathic “pins-and-needles” pain in the radial 3.5 digits. Moreover, these symptoms commonly affect both hands, including the thumb and radial fingers, with paresthesia potentially extending along the median nerve to the forearm, and even involving the ulnar digits [25].

Overall, the introduction of ICIs has led to significant advancements in cancer treatment, providing new hope and improved outcomes for patients in neoadjuvant, adjuvant, and metastatic settings [26,27].

## 2. Rare and Exclusive Events

### 2.1. Carpal Tunnel Syndrome Associated with Atezolizumab in a Male with Non-Small-Cell Lung Cancer

In March 2019, a 79-year-old male patient presented to the emergency department with persistent monthlong hemoptysis after being referred by his family doctor. He had been experiencing pain on the right side of his chest 3 months prior and had also reported losing 4 kg over the previous 3 weeks. He had a medical history of benign prostatic hyperplasia (BPH) and a 30-pack-year (PY) smoking history. Almost 10 years prior to this he suffered a myocardial infarction, subsequently treated with aspirin, an angiotensin-converting enzyme inhibitor (ACEi), and a beta-blocker. He also had a family history of RCC, as his sister had been diagnosed at the age of 79.

There were no abnormalities noted upon physical examination, including cardiovascular and pulmonary evaluation, and the electrocardiogram (ECG) was within normal ranges. No abnormalities were found in the routine laboratory investigations, including the biochemical profile and complete blood count. A computed tomography scan of the chest (CT) revealed a 7 × 3.5 cm endobronchial mass obstructing the right lower lung (RLL). Bronchoscopy was performed, and a biopsy of the RLL mass and hilar lymph nodes (LN) was obtained. The pathological analysis indicated that the mass was a moderately differentiated squamous cell carcinoma originating from the lung, and the LN that were checked were normal. Magnetic resonance imaging (MRI) of the head did not show any evidence of metastatic disease, and a positron emission tomography–computed tomography (PET-CT) scan revealed increased metabolic activity in the RLL mass and the right mediastinal area.

The cancer patient was diagnosed with stage IIB (T3-N0-M0) squamous cell carcinoma. Molecular testing showed programmed death ligand 1 (PD-L1) staining over 50%, negative for EGFR and BRAF mutations, and negative for ALK rearrangement.

A comprehensive multi-disciplinary meeting involving radiologists, cardiothoracic surgeons, and oncologists led to the determination that the patient’s optimal course of action would be systemic pre-operative (neoadjuvant) treatment. Our patient started a neoadjuvant therapy, which consisted of a combination of carboplatin (area under the curve (AUC) of 5) and atezolizumab (administered at a dose of 1200 mg) on day 1, every 3 weeks, plus nab-paclitaxel (Abraxane™) (80 mg per m^2^) on days 1, 8, and 15.

After completing two cycles of this treatment, the patient began experiencing pain in both wrists, with the right side more severely affected. This symptom persisted, and the patient was readmitted to undergo a chest CT scan after the third cycle. The results revealed a very favorable response, demonstrating a reduction in the tumor size to 1.1 cm in its largest dimension. Subsequently, in August 2019, the patient underwent a surgical resection procedure, known as a robotic lobectomy (RL), with the subsequent pathological analysis confirming that the surgical margins were free from disease.

Following this procedure, the patient’s treatment plan consisted of continuing atezolizumab monotherapy on day 1 every 3 weeks. After completing four cycles of this therapy, however, the patient reported experiencing severe pain accompanied by mild edema in both wrists, with the right side once again being more severely affected. Consequently, the treatment was halted, and the patient was reevaluated with head and chest CT scans. The results of these scans did not lead to a justification of the patient’s worsening clinical symptoms. As a result, the patient was referred to specialists in neurology and neurosurgery for further consultation.

**Figure 1:**
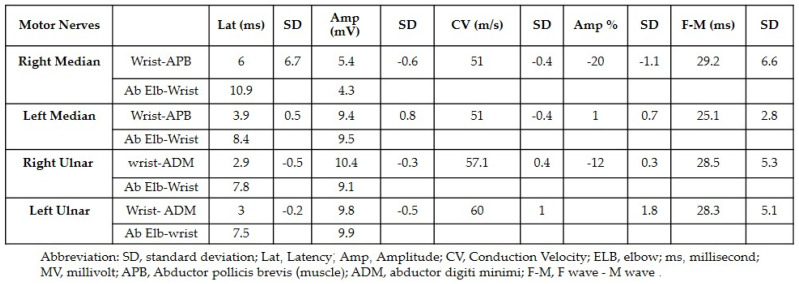
The results of the nerve conduction velocity showing the prolonged distal sensory latency observed in the left median nerve.

**Figure 2:**
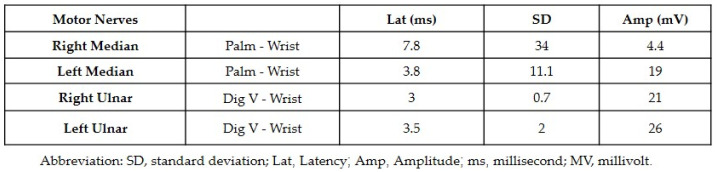
The results of the electromyography showing the motor latency, low snaps, and borderline compound muscle action potential test.

Both neurology and neurosurgery consultants observed that the patient exhibited bilateral median and ulnar nerve palsies, while the rest of the neurologic examination yielded normal results. The leading suspicion was severe carpal tunnel syndrome (CTS). Electromyography (EMG) and nerve conduction velocity (NCV) tests were performed, which indicated prolonged distal sensory and motor latency, low snaps, borderline compound muscle action potential (CMAP) test, and F waves in the right median nerve. Additionally, prolonged distal sensory latency was observed in the left median nerve in the NCV. These EMG findings were consistent with severe CTS in the right hand and mild CTS in the left hand (Figure 1 and Figure 2).

After being referred to an orthopedic surgeon, the patient underwent open surgery on the right hand. Specifically, bisection of the carpal tunnel ligament was performed to relieve nerve compression. Despite this procedure, the neuropathy and pain persisted with a subsequent worsening of muscle weakness in the median and ulnar distribution. This patient’s neuropathy progressed despite being several weeks removed from the operation.

**Figure 3:**
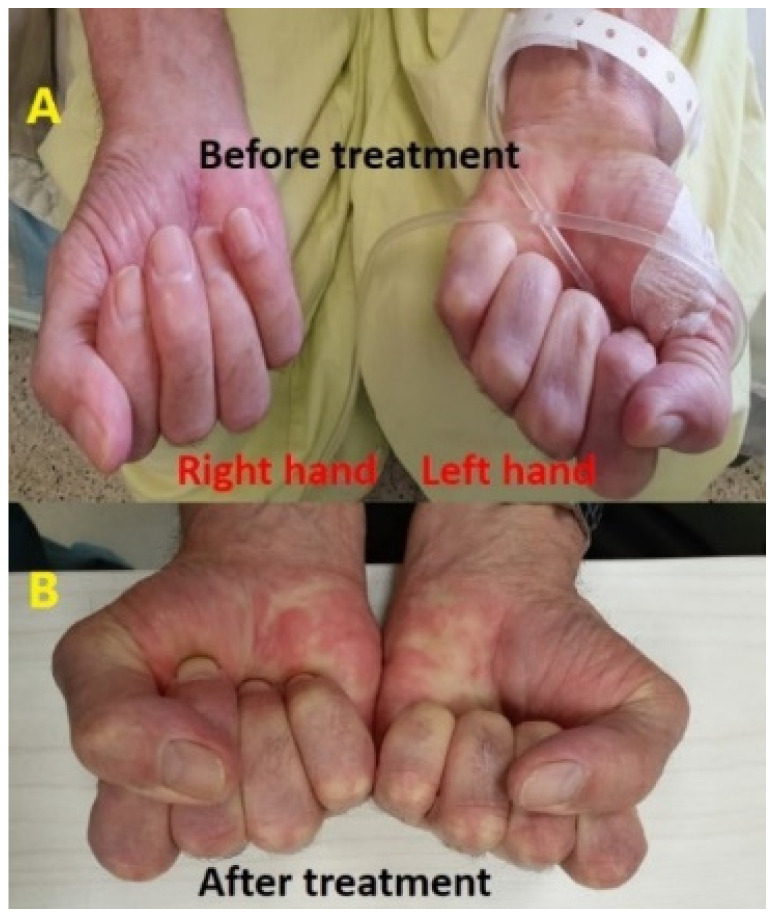
The difference in the difficulty of closing palms and maintaining hand grip was evident before (**A**) and after (**B**) IV-IG therapy, with a marked improvement observed in the hand grip strength and ease of closing palms following the treatment.

Treatment with a course of steroids, including prednisone at a dosage of 1 mg per kg, was initiated for a span of one week but yielded no discernible improvement. Subsequently, the patient was admitted for therapeutic intervention (immunosuppressive therapy) consisting of intravenous immunoglobulins (IVIG) at a daily dosage of 0.4 mg per kg, administered over a period of five days. Notably, by the third day of this treatment regimen, a significant clinical improvement was evident in both hands, with motor and sensory functions fully restored to normal levels (Figure 3). Later, the patient continued receiving IVIG as a maintenance therapy monthly for 4 months. All symptoms were subsequently resolved and no additional irAEs were observed. The patient remains in follow-up with no evidence of disease recurrence (last consultation in March 2023). 

### 2.2. Carpal Tunnel Syndrome Associated with Nivolumab in a Female with Renal Cell Carcinoma

In May 2015, a female 70-year-old patient without any notable medical history or family history of cancer was referred to the emergency department by her family doctor. During the previous 2 months, she had been experiencing discomfort and escalating left flank pain. The patient underwent an abdominal CT scan which revealed a mass on the left kidney involving the left renal vein and LN. To further investigate the condition, a PET-CT scan was performed which confirmed the findings from the abdominal CT scan. Distant metastasis was also ruled out with the PET-CT scan results. In July 2015 the patient had a left radical nephrectomy. A histopathologic analysis of the excised tissue indicated RCC with involvement of the renal vein and five out of eight LN. The patient was classified as stage III.

Nevertheless, the patient rejected all offers of treatment or subsequent care. In April 2016 she manifested with extradural and intracranial metastases, prompting her admission for an excisional biopsy of the extradural metastasis. This procedure conclusively verified the presence of metastatic RCC. A subsequent PET-CT scan unveiled an increased metabolic activity in the hilum of the left lung (measuring 1.7 cm), the area of the left mediastinum, peripheral nodules in both lungs (measuring 0.6 cm), the left adrenal gland, right femoral neck, and proximal left humerus. The cancer was then classified as stage TX-N0-M1 (stage IV).

Pazopanib treatment was started at an oral dose of 800 mg per day. However, in October 2017, during a routine follow-up, a total body CT (TB-CT) scan revealed disease progression in the lungs and mediastinum, indicating a worsening of the condition, necessitating further treatment.

The patient exhibited a favorable radiological response following the inclusion of nivolumab therapy at a dosage of 3 mg per kg. However, in March 2020 the patient started experiencing severe pain in both wrists. She reported more severe symptoms on the right side. A multidisciplinary conference involving an oncologist and immunologist concluded that the patient was likely experiencing CTS secondary to ICI therapy (nivolumab specifically). Nivolumab treatment was therefore ceased, and the patient was referred for a neurologic consultation.

**Figure 4:**
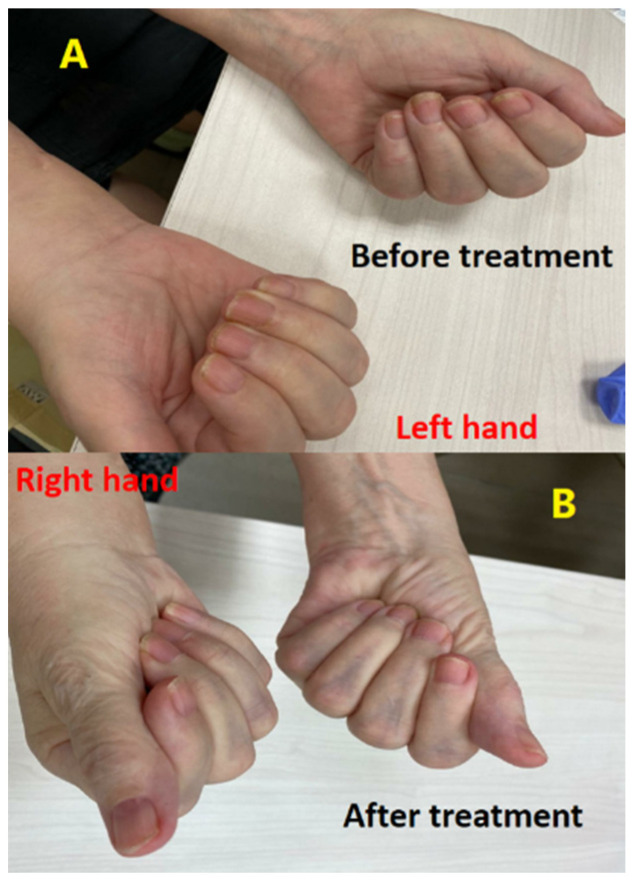
The difference in the difficulty of closing palms and maintaining hand grip was evident before (**A**) and after (**B**) corticosteroid therapy, with a noticeable improvement observed in hand grip strength and ease of closing palms following the treatment.

It was determined through this consultation that the patient had bilateral median and ulnar nerve palsies. Corticosteroid treatment was started with prednisone at a 1 mg per kg dosage given the suspicion of CTS associated with nivolumab therapy. There was only minor improvement observed following one week of treatment, prompting an increase in the prednisone dosage to 2 mg per kg. She reported an improvement in pain symptoms two weeks after increasing the dose. Subsequent follow-up neurologic examinations indicated improvement of the ulnar and median nerve palsies, (Figure 4). The patient continued a maintenance dose of 10 mg daily prednisone with rechallenging nivolumab. Although no additional irAEs were observed, the patient died in February 2021 as a result of disease progression.

### 2.3. Carpal Tunnel Syndrome Associated with Nivolumab in a Male with Melanoma

In 2012, a 71-year-old male patient underwent resection for stage I melanoma (left shoulder). In February 2019, during a dermatologic follow-up, two new skin lesions were discovered on the left upper back, measuring 4.2 cm and 2.2 cm in diameter, respectively. Subsequently, a PET-CT scan showed an increased metabolic activity in the left upper back.

A multidisciplinary meeting involving a plastic surgeon, oncologist, and radiologist concluded that these findings indicated a recurrence of melanoma. As a result, it was recommended to perform a comprehensive excision of the affected area. The subsequent histopathologic analysis confirmed the presence of oligo-metastatic melanoma. The pathological stage was identified as TX-NX-M1 (stage IV), and molecular testing revealed a wild-type BRAF genotype. To treat the oligo-metastatic disease, the patient received intravenous immunotherapy with nivolumab at a dosage of 3 mg per kg on the first day, every two weeks for twelve cycles. Throughout this treatment period, PET-CT scans showed no evidence of disease.

It was decided to increase the dosage of nivolumab to a fixed dose of 480 on the first day (due to the COVID-19 pandemic), allowing for therapy every four weeks. Nevertheless, the patient began experiencing severe discomfort in both wrists after two cycles of treatment at the elevated dosage. This discomfort escalated and was accompanied by increased weakness by the third cycle.

Considering the experiences from the previous two cases described above, nivolumab was discontinued. The patient was then referred for consultations with an orthopedic surgeon and a neurologist due to the suspicion of CTS. Examination by the neurologist confirmed bilateral median and ulnar nerve neuropathy. Imaging studies using ultrasound of the wrists and plain X-ray revealed fluid accumulation between mildly swollen flexor tendons, indicating tenovaginitis (tenosynovitis, trigger finger).

Corticosteroid treatment was started with prednisone at a dosage of 1.5 mg per kg. After ten days of therapy, a noteworthy clinical improvement was evident in both hands. Subsequently, in July 2020, he underwent a TB-CT scan for follow-up, which revealed no signs of disease. The patients remained in follow-up with pregabalin and non-steroidal anti-inflammatory drugs with minimal CTS symptoms. 

### 2.4. Carpal Tunnel Syndrome Associated with Ipilimumab plus Nivolumab in a Male with Melanoma

In March 2021, a 68-year-old male patient with no significant medical or family history presented to our cancer center after being referred by a dermatologist due to melanoma. The patient described several new lesions resembling sebaceous cysts that appeared on his upper back almost 4 months prior to the diagnostic biopsy. The maximal diameter of these lesions was 3.6 cm. Subsequently, a PET-CT scan revealed increased metabolic activity in several areas of the upper back region bilaterally and the lateral left chest area. Lung nodules were observed as well as an increased metabolic activity and diameter of the cervical lymph nodes bilaterally.

A multidisciplinary conference involving an oncologist, plastic surgeons, dermatologists, and a radiologist concluded that these findings were indicative of metastatic melanoma. The cancer was then classified as stage TX NX M1 (stage IV). Molecular testing revealed a BRAF wild-type genotype.

To address the metastatic disease, the patient received intravenous immunotherapy with nivolumab at a dosage of 1 mg per kg plus ipilimumab at a dosage of 3 mg per kg on day 1, every 3 weeks for four cycles. Throughout this treatment period, PET-CT scans showed a partial response. Therefore, nivolumab at a dosage of 3 mg per kg every 2 weeks was continued. In November 2022 (after 23 cycles of nivolumab), the patient suffered from a grade 2 rash and intense pain and weakness in both wrists. A multidisciplinary team consisting of an oncologist, rheumatologist, immunologist, and neurologist concluded that the patient’s symptoms were related to nivolumab therapy.

**Figure 5:**
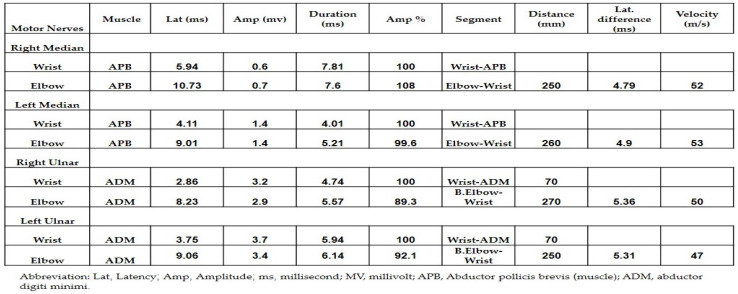
The results of the nerve conduction velocity showing CTS.

Treatment was discontinued and corticosteroid therapy was initiated with prednisone at a dosage of 1.5 mg per kg. After one week of treatment, the patient reported no rash and a reduction in pain in both wrists, with weakness persisting. At this stage, the patient underwent EMG for both hands which revealed motor latency and prolonged distal sensory latency in the left and right median nerve. The CMAP test showed low snaps with severe proximal and distal time lags, severe CTS in the right hand, and mild CTS in the left hand (Figure 5). 

A multidisciplinary team consisting of an oncologist, rheumatologist, immunologist, and neurologist subsequently concluded that the patient’s CTS was induced by nivolumab therapy and resistant to corticosteroid therapy. Therefore, the patient started methotrexate therapy (15 mg weekly). The patient reported a significant improvement in pain and weakness the following month. Therefore, it was decided to resume the nivolumab therapy with continued methotrexate. In February 2023 the patient underwent a TB-CT scan which revealed a complete radiological response. Finally, it was decided to leave the patient for follow-up. The last PET-CT from July 2023 showed similar findings and the patient continued methotrexate therapy at the same dose.

## 3. The ICI Mechanisms of Action

Conventional T cells (Tcon) employ two mechanisms to target tumor cells. The first mechanism involves antigen-specific signaling through cognate T cell receptors (TCRs) and peptide-MHC (pMHC), while the second mechanism relies on the ligation of nonspecific coreceptor signaling. These receptors can either be costimulatory or coinhibitory, enhancing or attenuating T cell responses, respectively. CD28 serves as an important costimulatory receptor, whereas CTLA-4 and PD-1 function as coinhibitory receptors.

CTLA-4 (CD152), a member of the B7/CD28 family, facilitates immunosuppression by indirectly inhibiting signaling via the CD28 costimulatory receptor. Its higher affinity for CD80 (B7-1) and CD86 (B7-2) results in competitive inhibition of CD28 costimulation during T cell priming, reducing the release of proinflammatory cytokines like IL-12 and cytotoxic enzymes such as perforin and granzyme B [13,14,28,29,30,31]. CTLA-4 also mediates the endocytosis of CD80 and CD86 on antigen-presenting cells (APCs), reducing their availability for CD28. Consequently, the activation threshold of T cells is increased, leading to diminished immune responses to low avidity TCR–pMHC interactions, including self- and tumor-associated antigens (TAAs). Blockade of CTLA-4 with a monoclonal antibody has therefore been explored as a therapeutic strategy to enhance anti-tumor immunity [11,13,32,33,34].

Programmed death 1 (PD-1) is an inhibitory transmembrane protein expressed in T cells, B cells, natural killer cells (NKs), and myeloid-derived suppressor cells (MDSCs). It interacts with its ligands, PD-L1 (also known as B7-H1) and PD-L2 (B7-H2), which are expressed in various tissue types, including tumor cells and hematopoietic cells. The PD-1-PD-L1/2 interaction leads to several inhibitory mechanisms, including inhibition of tumor cell apoptosis, exhaustion of peripheral effector T cells, and conversion of effector T cells into regulatory T cells (Tregs). By blocking the PD-1 pathway, T cell anti-tumor activity can be enhanced, promoting immune surveillance and targeted tumor cytotoxicity [35,36,37].

## 4. Immune Checkpoint Inhibitors Therapies

### 4.1. PDL-1 Inhibitors

#### 4.1.1. Atezolizumab

Atezolizumab is a PD-L1 inhibitor with FDA approval for the treatment of various cancer types, including NSCLC, urothelial carcinomas (UC), MM, triple-negative breast cancer, and hepatocellular carcinoma (HCC).

Some of the commonly observed adverse events associated with atezolizumab include vomiting, dyspnea, diarrhea, hypothyroidism, headache, hyperthyroidism, rash, nausea, cough, fatigue, decreased appetite, alopecia, and constipation. While these side effects are more frequently reported, it is important to note that severe and potentially life-threatening events caused by atezolizumab are rare. Such potentially life-threatening events may include drug rash with eosinophilia, toxic epidermal necrolysis, systemic symptoms, as well as Stevens–Johnson syndrome, and acute generalized exanthematous pustulosis (Table 1). Atezolizumab has not been previously reported to be associated with CTS [13,14,38].

#### 4.1.2. Durvalumab

Durvalumab is also a PD-L1 inhibitor. It has received FDA approval for the treatment of several types of cancer, such as HCC, NSLC, and TCC. The commonly reported irAEs of durvalumab were pneumonitis, dyspnea, diarrhea, endocrinopathies, cough, constipation, hypothyroidism, and hyperthyroidism (Table 1). Durvalumab has not been previously reported to be associated with CTS [13,14,39].

#### 4.1.3. Avelumab

Avelumab is also a PD-L1 inhibitor. It has obtained FDA approval for the treatment of numerous cancer types, such as advanced refractory urothelial bladder carcinoma, in combination with axitinib in metastatic RCC, and metastatic Merkel cell carcinoma (MCC) (Table 1) [40,41]. Avelumab has been previously reported to be associated with CTS in only one single case of a female with Merkel cell carcinoma [42].

### 4.2. PD-1 Inhibitors

#### 4.2.1. Pembrolizumab

Pembrolizumab is a PD-1 inhibitor. Unlike typical IgG antibodies, pembrolizumab does not activate antibody-dependent cellular cytotoxicity. It has obtained FDA approval for the treatment of numerous cancer types, such as MCC, UC, NSCLC, different types of squamous cell cancers (SCC), MM, and RCC. It was observed to potentially induce irAEs in around 70% of patients. The irAEs due to pembrolizumab typically include diarrhea, hypothyroidism, rash, hyperthyroidism, fatigue, constipation, pruritus, fever, cough, and myalgia (Table 1) [13,14,43,44]. Pembrolizumab has been previously reported to be associated with CTS in four females and two males treated for MM. Additionally, there is a single reported case of a male patient who developed CTS while being treated with pembrolizumab for squamous cell carcinoma of the face (skin) [42].

#### 4.2.2. Nivolumab

Nivolumab is also a PD-1 inhibitor which, like pembrolizumab, does not lead to antibody-dependent cellular cytotoxicity. It has obtained FDA approval for the treatment of numerous cancer types, such as colorectal cancer, NSCLC, MM, esophageal SCC, RCC, UC, mesothelioma, HCC, and lymphoma. The irAEs usually include hyperthyroidism, vomiting, fever, hypothyroidism, dyspnea, constipation, nausea, diarrhea, rash, headache, cough, abdominal pain, and pruritus. 

It is recognized that the onset of these nivolumab irAEs can manifest even after completing the treatment. This delayed onset could be attributed to the enduring impact of nivolumab, which might lead to the deactivation of PD-1 receptors for a span of approximately three months. Notably, the concurrent usage of nivolumab and ipilimumab has been shown to result in a heightened incidence of AEs in comparison to the utilization of nivolumab alone (Table 1) [13,14,44,45]. Nivolumab has been previously reported to be associated with CTS in two females who were treated for MM [42].

#### 4.2.3. Cemiplimab

Cemiplimab, another PD-1 inhibitor, works similarly to nivolumab and pembrolizumab. It has obtained FDA approval for the treatment of numerous cancer types, such as NSCLC, SCC, and basal cell carcinoma. In patients receiving cemiplimab, the most common irAEs may include rash, nephritis, hepatitis, pneumonitis, colitis, hypothyroidism, hyperthyroidism, and pruritus. Among patients taking cemiplimab, the predominant irAEs may include pruritus, musculoskeletal pain, fatigue, pneumonitis, diarrhea, rash, nephritis, colitis, and hepatitis (Table 1). Cemiplimab has not been previously reported to be associated with CTS [13,14,34,46].

### 4.3. CTLA-4 Inhibitors

#### Ipilimumab

Ipilimumab is a fully humanized recombinant monoclonal antibody that predominantly targets CTLA-4. It has obtained FDA approval for the treatment of numerous cancer types, such as colorectal cancer, RCC, NSCLC, HCC, and MM. Ipilimumab is infrequently employed as monotherapy and is typically administered in conjunction with nivolumab. This combination has demonstrated increased response rates compared to the use of ipilimumab alone. However, it is important to note that the combination therapy is associated with a higher incidence of AEs compared to ipilimumab monotherapy.

Unlike traditional cytotoxic chemotherapy, the mechanism of action of ipilimumab involves stimulating the immune system, which means that it generally does not lead to side effects like bone marrow suppression. Nevertheless, it is important to note that 90% of patients might encounter irAEs of ipilimumab. These irAEs can manifest as pruritus, neuropathy, endocrinopathy, hepatitis, rash, dermatitis, fatigue, colitis, and diarrhea (Table 1). Ipilimumab has not been previously reported to be associated with CTS [13,14,47], although it has been previously reported to be associated with CTS in a male patient who received a combination therapy of ipilimumab and nivolumab for the treatment of MM [42].

## 5. Discussion

Carpal tunnel syndrome (CTS) is a prevalent condition characterized by the compression of the median nerve, making it the most common nerve compression neuropathy affecting the global adult population. The development of CTS can be attributed to various pathological conditions that lead to a reduction in the cross-sectional area of the carpal tunnel or an expansion of the components within the carpal tunnel. Over the past decade, the incidence of CTS has been on the rise.

CTS predominantly affects women, with a three-fold higher prevalence compared to men. The condition is more commonly observed in women and men between the ages of 30 and 40, and 60 and 80, respectively. Bilateral involvement occurs in approximately 60% of CTS cases. Several constitutional factors and comorbidities contribute to the development of CTS. High body mass index, advanced age, pregnancy, menopause, and female gender are significant constitutional risk factors. Clinical comorbidities such as rheumatoid arthritis, hypothyroidism, obesity, and diabetes mellitus are also associated with CTS. In cases where no specific cause can be identified, the condition is classified as idiopathic.

Clinically, CTS patients exhibit numbness, pain, and tingling sensations within the distribution of the median nerve in the hand or arm. These symptoms may be accompanied by weakness and atrophy of the muscles located in the thumb region, resulting in a loss of hand strength. Sensory changes confined to the median nerve distribution in the hand, as well as the presence of Tinel’s and Phalen’s signs, are crucial indicators for making a clinical diagnosis of CTS [48,49,50]. 

ICIs hold great promise for achieving long-term and even complete responses in certain patients, marking a significant advancement in cancer treatment. However, it is essential to acknowledge that not all patients respond favorably to these therapies, and there are challenges in assessing the true clinical impact of immune-oncology (IO) drugs. One of the major hurdles is distinguishing between genuine treatment responses and atypical patterns such as pseudoprogression or hyperprogression. Pseudoprogression refers to a temporary increase in tumor size or the appearance of new lesions on imaging, which may initially mimic disease progression but is later followed by a positive response to the treatment. On the other hand, hyperprogression is characterized by an accelerated tumor growth rate after ICI treatment, which can be detrimental to patients. Unlike conventional oncology therapies, where higher drug doses may lead to increased toxicity, the side effects of ICIs do not appear to be directly linked to the drug dose. Thus, reducing the drug dose is unlikely to prevent immune-related toxicities from occurring. This poses a significant consideration for physicians when managing patients undergoing ICI treatments. Vigilant monitoring for immune-related toxicities is crucial during ICI therapy. These toxicities can be severe and may require permanently discontinuing treatment, especially when using combination ICI strategies. Balancing the potential benefits of combination therapies with the risk of increased toxicity is a complex decision that oncologists must carefully weigh for each patient [51,52,53,54]. However, the remarkable efficacy of these immune checkpoint inhibitors comes with the risk of immune-related toxicities. These toxic effects occur as a result of disrupting the mechanisms that maintain self-tolerance, leading to immune responses that resemble autoimmune reactions [52,53].

In different phase I to III clinical trials it has been reported that the occurrence of any grade neurological irAEs, which may include unspecific symptoms such as headache and dizziness, varied between 0% and 27% for the anti-PD-1 and the anti-CTLA-4 treatments. The median incidence of these neurological irAEs was almost 3.8% in patients treated with ipilimumab and tremelimumab and 6.1% in patients who received anti-PD-1 treatment. This finding contrasts with the fact that irAEs are generally more common during anti-CTLA-4 therapies. Notably, higher incidence rates of neurological irAEs are observed when combination therapy with both anti-CTLA-4 and anti-PD1 treatments is administered. However, the incidence of severe grades of irAEs (grades 3 to 4) and neurological irAEs in clinical trials remained below 1%, even among patients who received combination therapy [52,53,54,55]. The neurological irAEs could present at different times, with the occurrence typically varying between three and thirteen weeks after the initiation of ICI therapy. They can affect both the central nervous system (CNS) and the peripheral nervous system (PNS), leading to a diverse spectrum of neurological complications. Involvement of the CNS may result in conditions such as encephalitis (inflammation of the brain) and aseptic meningitis (inflammation of the membranes surrounding the brain and spinal cord). Conversely, the PNS can also be impacted, leading to conditions like acute immune demyelination, cranial nerve neuropathies, myasthenic syndromes, myositis (inflammation of the muscles), polyneuropathy, and chronic immune demyelinating polyneuropathy. Among neurological irAEs, neuromuscular disorders constitute the most frequently documented, accounting for around 50% of cases. These disorders predominantly encompass myositis, myasthenia gravis, demyelinating polyradiculoneuropathy, and overlapping syndromes.

Managing neurological irAEs hinges on their severity, classified on a scale from 1 to 4. In instances of grade 1 neurological symptoms, ICI treatment is typically continued, albeit with vigilant monitoring for any signs of deterioration. In the event of grade 2 neurological symptoms, ICI treatment should be halted, and the patient should initiate a regimen of oral or intravenous corticosteroids (such as methylprednisolone) to regulate the immune response. For grades 3 or 4 neurological symptoms, more intensive immune modulation may be essential, in conjunction with corticosteroid therapy. In specific scenarios, corticosteroids might be substituted with alternative treatments like intravenous immunoglobulin (IVIG), plasma exchange, or selective separation to address the severity of the immune-related reaction [52,53,54,55,56]. 

The occurrence of neurological AE associated with ICI treatment has expanded to include conditions like painful brachial plexus neuritis. Understanding and identifying these uncommon adverse effects is crucial for timely intervention and appropriate management [57].

One of the best-characterized actions of neuroactive steroids in PNS is the regulation of myelin production. Namely, neuroactive steroids exert their physiological actions on the expression of myelin proteins as well as transcription factors involved in the regulation of myelination. This effect relies on classical and non-classical steroid receptors. Indeed, progesterone and its metabolites modulate the expression of myelin proteins of the PNS, such as myelin basic protein, myelin proteolipid protein, glycoprotein zero (P0), and peripheral myelin protein as well as myelin formation. Neuropathic pain is a chronic pain condition that may arise after an injury or disease affecting the somatosensory system. Neuroactive steroids control important aspects of the development, activity, and plasticity of the nervous system [58,59,60,61,62,63].

In addition to pain-reducing effects in different neuropathic pain models, steroids are able to restore biochemical, functional, and morphological parameters after peripheral nerve injury [61,62,63].

For example, in the chronic constriction injury (CCI) model, 14 days of PROG treatment initiated 12 days after the injury improves the electrophysiological changes in motor and sensory conduction velocity, thermal hyperalgesia, and mechanical allodynia produced by injury. Moreover, Coronel and colleagues reported that timely PROG administration reduces the number of glial-fibrillary-acidic-protein-positive cells and avoids the development of mechanical and thermal allodynia, as it is administered early enough to block the post-SCI inflammatory cascade [58,64,65]. 

The significant relief and improvement of symptoms following treatment with steroids and IVIG, along with the bilateral occurrence, strongly suggest that the symptoms are a result of a neurologic immune-related AE rather than an independent coincidental event. Although the exact immune-mediated mechanism remains unclear, it is conceivable that peripheral T cell dysregulation plays a substantial role. In managing neurologic irAEs, a common approach involves discontinuing ICIs and initiating high-dose corticosteroid treatment. This strategy has demonstrated beneficial outcomes in the majority of cases. However, the decision to incorporate corticosteroid-sparing agents during the course of irAE treatment should be carefully considered on an individual basis, taking into account factors such as the severity of the irAE and the overall clinical condition of the patient.

As mentioned previously, and based on the available knowledge, the reported association of atezolizumab with CTS appears to be a rare occurrence in oncologic patients, particularly those with lung cancer. Similarly, in the case of a patient diagnosed with RCC, the development of CTS induced by nivolumab is also an uncommon finding. 

Further studies are necessary to determine whether an increased susceptibility to irAEs is associated with similar genetic alterations or if the pathogenesis of irAEs is multifaceted. Comparing the incidence of irAEs across different clinical trials may present challenges due to the use of diverse classification criteria for AEs and variations in the terminology and reporting frequency thresholds for irAEs. Moreover, discrepancies in follow-up periods and treatment dosages within and between studies can impact the detection rate of irAEs.

## 6. Conclusions

Optimal management of irAEs necessitates a careful balance between controlling the AEs while preserving the anti-tumor efficacy of ICIs. Closely monitoring patients who receive ICI therapy is crucial to detect early signs of neurological toxicities. Healthcare providers can optimize patient outcomes and minimize the potential complications associated with neurologic irAEs by recognizing symptoms promptly and initiating appropriate treatments. This proactive approach ensures that patients can continue to benefit from the proven anti-tumor effects of ICIs while effectively managing any AEs that may arise.

## Figures and Tables

**Table 1:** Immune checkpoint inhibitors: indications, and adverse effects.

Therapy	ICI Therapies	Approval	Cancer Diagnoses	Predominant Adverse Events
Ipilimumab	CTLA-4 inhibitor	2011	CRC, Melanoma, NSCLC, HCC, RCC, and SCLC	Pruritis, fatigue, rash, diarrhea, transaminitis, and colitis
Pembrolizumab	PD-1 inhibitor	2014	Melanoma, SCC, lung cancer, lymphomas, cancers high in MSI, urothelial carcinoma, MMR-deficient cancers, gastric cancers, cervical cancers, HCC, Merkel cell carcinoma, esophageal cancers, RCC, endometrial carcinoma, tumor mutational burden-high cancer, and triple-negative breast cancer	Myalgia, fatigue, decreased appetite, diarrhea, constipation, rash, fever, cough, pruritis, and nausea
Nivolumab	PD-1 inhibitor	2014	NSCLC, Melanoma, urothelial carcinoma, RCC, malignant pleural mesothelioma, HCC, classic Hodgkin lymphoma, HNSCC, CRC, and SCC of the esophagus	Fatigue, rash, diarrhea, and pruritis
Cemiplimab	PD-1 inhibitor	2018	Cutaneous SCC, basal cell carcinoma, and NSCLC	Nephritis, pneumonitis, hepatitis, rash, hypothyroidism or hyperthyroidism, pruritus, and colitis
Atezolizumab	PDL-1 inhibitor	2016	NSCLC, Urothelial carcinoma, SCLC, HCC, melanoma, and triple-negative breast cancer	Fatigue, nausea, vomiting, cough, dyspnea, constipation, decreased appetite, diarrhea, alopecia, or headache and rash
Durvalumab	PDL-1 inhibitor	2017	NSCLC and Urothelial carcinoma	Fatigue, constipation, UTIs, edema, pneumonitis, dyspnea, rash, cough, and nausea

Abbreviations: ICI, immune checkpoint inhibitors; SCLC, small-cell lung cancer; RCC, renal cell carcinoma; SCC, squamous cell carcinoma; CRC, colorectal cancer; NSCLC, non-small-cell lung cancer; HNSCC, head and neck squamous cell carcinoma; HCC, hepatocellular carcinoma; MMR, mismatch repair; MSI, microsatellite instability; UTIs, urinary tract infections; PDL-1, programmed death ligand 1; PD-1, programmed cell death 1.

## Data Availability

Data are contained within the article or are available from the authors upon reasonable request.
